# Rationale, design, and implementation protocol of the Dutch clinical practice guideline Pain in patients with cancer: a cluster randomised controlled trial with short message service (SMS) and interactive voice response (IVR)

**DOI:** 10.1186/1748-5908-6-126

**Published:** 2011-12-06

**Authors:** Nienke te Boveldt, Yvonne Engels, Kees Besse, Kris Vissers, Myrra Vernooij-Dassen

**Affiliations:** 1 Department Anesthesiology, Pain and Palliative Medicine, Radboud University Nijmegen Medical Centre (RUNMC), Nijmegen, 6500 HB, The Netherlands; 2 Department IQ Healthcare/Department of Primary and Community Care, Radboud University Nijmegen Medical Centre (RUNMC)/Kalorama foundation, Nijmegen, 6500 HB, The Netherlands

## Abstract

**Background:**

One-half of patients with cancer have pain. In nearly one out of two cancer patients with pain, this was undertreated. Inadequate pain control still remains an important problem in this group of patients. Therefore, in 2008 a national, evidence-based multidisciplinary clinical practice guideline 'pain in patients with cancer' has been developed. Yet, publishing a guideline is not enough. Implementation is needed to improve pain management. An innovative implementation strategy, Short Message Service with Interactive Voice Response (SVS-IVR), has been developed and pilot tested. This study aims to evaluate on effectiveness of this strategy to improve pain reporting, pain measurement and adequate pain therapy. In addition, whether the active role of the patient and involvement of caregivers in pain management may change.

**Methods/design:**

A cluster randomised controlled trial with two arms will be performed in six oncology outpatient clinics of hospitals in the Southeastern region of the Netherlands, with three hospitals in the intervention and three in the control condition. Follow-up measurements will be conducted in all hospitals to study the long-term effect of the intervention. The intervention includes training of professionals (medical oncologists, nurses, and general practitioners) and SMS-IVR to report pain in patients with cancer to improve pain reporting by patients, pain management by medical oncologists, nurses, and general practitioners, and decrease pain intensity.

**Discussion:**

This innovative implementation strategy with technical tools and the involvement of patients, may enhance the use of the guideline 'pain in patients with cancer' for pain management. Short Message Service alerts may serve as a tool to support self-management of patients. Therefore, the SMS-IVR intervention may increase the feeling of having control over one's life.

**Trail registration:**

Netherlands Trial Register (NTR): NTR2739

## Background

Pain is a major healthcare problem for patients with cancer [1] and is one of the most frequently feared symptoms [2,3]. In 2007, in a Dutch study 64% of patients with metastatic, advanced, or terminal disease [4], 59% of those on anti-cancer treatment and 33% of patients after curative treatment experienced pain [4]. Often, pain control is inadequate [2-9]. In 2007, Deandrea *et al*. demonstrated that pain in nearly one-half patients with cancer is undertreated [10]. As illustrated by the high prevalence of pain, for most patients acceptable pain reduction has not yet been reached. Up to now, no hospital-wide intervention has yet improved the treatment of pain in general [11].

A key barrier to adequate treatment of pain is ineffective communication between patients and healthcare providers about their pain [12,13]. Patients often consider information they receive from providers to be unclear [14,15]. Generally, patients lack knowledge about pain and pain management [16,17]. Several studies show that informing and educating the patient about treatment of cancer pain reduces pain intensity [18-21].

Professionals do not ask their patients systematically about their pain [22,23]. Moreover, patients seem to be reluctant to talk about their pain or to ask for pain medication [24-26] for a variety of reasons, such as concerns about addiction, tolerance, desire to please providers, and fear that reporting pain will take the physician's time away from the treatment of their cancer [27,28].

One further aspect of underreporting pain concerns assessment and documentation. There is evidence that careful and regular, systematic assessment of pain improves the perception of physicians and nurses concerning cancer pain and enhances the quality of pain management [29,30].

Healthcare providers tend to show a lack of attention to and knowledge about pain management [29,31-33] and consequently do not always treat pain according to specific guidelines [31,32]. This has been regarded as one of the main factors causing inadequate pain relief in cancer patients [29,34,35]. For these patient- and professional-related reasons, inadequate treatment of cancer pain persists, despite decades of efforts to provide clinicians with information on analgesics and pain-relieving techniques [36-42], and despite the availability of evidence-based guidelines on cancer pain [43].

The prevailing principle for treatment of cancer pain is the World Health Organization (WHO) three-step pain ladder, published in 1986 [42]. If this guideline is well applied, it is possible to achieve adequate pain relief in 70 to 90% of cancer patients [44-47].

Based on this pain ladder, a more detailed European recommendation for the use of morphine and alternative opioids has been published by the European Association for Palliative Care (EAPC)[48]. The final version of the 'Evidence-based guidelines for the use of opioids analgesics in the treatment of cancer pain: The EAPC recommendations' is in development [49].

The Dutch guideline 'Pain in patients with cancer'[50] is one of the most recent guidelines on this topic in Europe. It combines new insights and existing knowledge derived from evidence-based medicine. All relevant professional organizations of the Netherlands as well as the patient association have been involved in the development process. In a comparative study of European guidelines on this topic with the AGREE II instrument, this Dutch guideline appeared to have followed a good development process [51]. Yet, under-treatment of cancer pain may be partly caused by a lack of implementation of these clinical practice guidelines (CPGs)[10,52-54].

The present study aims to evaluate the implementation of the Dutch guideline 'Pain in patients with cancer'[50] to improve pain reporting, pain measurement, and hence pain control in patients with cancer and pain. A randomised controlled trial (RCT) with two arms will be performed in which professionals will be trained and Short Message Service with Interactive Voice Response (SMS-IVR) will be used to monitor and report pain.

Using Short Message Service (SMS) as a reminder and as tool to collect data on pain scores is innovative and promising [55]. Mobile phones are part of daily life; in 2009, nine out of ten Dutch inhabitants used a mobile phone [56]. SMS alerts have been used for asthma management [57-59], management of irritable bowel syndrome [60,61] management of diabetic patients [61] and recurrent pain in children aged 9 to 15 [62]. These studies concluded that SMS can serve as a tool to support self-management of patients. The use of mobile phone SMS alerts in the present study may be a way to encourage patient empowerment, because the patients' role in their pain management becomes more active. Empowerment has been defined by its absence of helplessness, or the feeling of having greater control over one's life [63].

We expect that SMS-IVR will increase the percentage of patients with cancer who receive adequate pain treatment and reduce pain intensity in patients with cancer, because pain will be measured systematically. In addition, patients are expected to become less reluctant to report pain and physicians will ask patient more frequently about pain.

The primary research question of the present study is: Will implementation of the Dutch guideline improve pain reporting, pain measurement, and adequate pain therapy?

A RCT will be implemented, with clustering based on number of beds and number of medical oncologists to increase comparability of hospitals and to reduce contamination [64]. Differences of the effectiveness of the intervention between subgroups are expected. Factors that may predict inadequate cancer pain treatment include gender, race, low education, a better physical condition without metastatic disease, and age [65]. This paper describes the aims and methods of an RCT to evaluate on effectiveness of implementation of the Dutch guideline to improve pain reporting, pain measurement, and adequate pain therapy. The results of this study will be published in several scientific papers.

## Methods/design

### Objectives/hypothesis

The primary objective of this RCT is to reduce pain intensity of patients with cancer. The secondary objectives are to improve knowledge of the guideline of oncologists, nurses, and general practitioners (GPs) to increase pain reporting by patients and professionals, to increase systematic pain measurement by medical specialists and nurses working at oncology outpatient clinics, and increase quality of life of patients.

It is hypothesized that this innovative implementation strategy--which includes use of technical tools, training of professionals, and patient involvement--may increase the use of the guideline for pain management in cancer patients, and consequently reduce pain intensity (individual level and cluster level) and increase pain management. SMS-IVR alerts may serve as a tool to support self-management of patients.

### Time frame

This study will be conducted from 2011 to 2015.

### Study design

**Figure 1:**
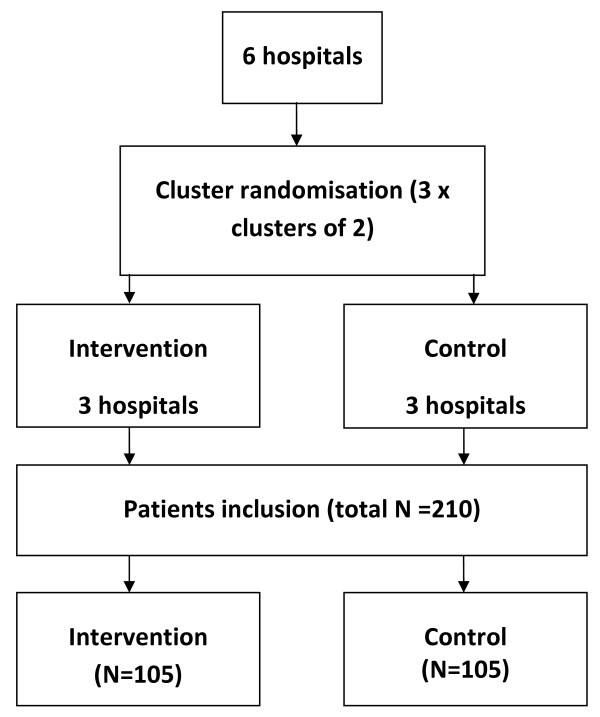
**Flowchart cluster randomisation of clinics**. Figure 1 shows the cluster randomisation of clinics. A cluster RCT with two arms will be performed in six oncology outpatient clinics of hospitals, with three hospitals in the intervention and three in the control condition. Clusters of hospitals will be determined based on number of beds and number of medical oncologists. We require 35 patients per hospital, a total of 210 patients.

A non-blinded cluster RCT, will be performed in six oncology outpatient clinics of hospitals in the Southeastern region of the Netherlands, with hospital as cluster. Stratified randomisation will be performed based on pairs of two comparable hospitals regarding number of beds and number of medical oncologists. For each pair, one hospital will be randomly allocated to the intervention condition and the other to the control condition. Allocation to the intervention or control condition will be done before start of the intervention period by asking a statistician to select three closed envelopes (Figure 1). The allocation was generated by an independent statistician. Chosen implementation strategies are:

1. Training of oncologists and nurses involved in cancer care on the most important aspect of the CPG comprising of three one-hour sessions, one main session at baseline, session two at month six, and the final session at month twelve (intervention arm).

2. Patients will receive SMS-IVR and personal advice by phone on how to reduce their pain if their pain rating is 5 or higher on a numeric rating scale (NRS) of 0 (no pain at all) to 10 (worst pain you can imagine) (intervention arm).

3. Patients will receive a leaflet on cancer pain of the Dutch Cancer Society (in both arms).

4. Oncologists and nurses will receive a leaflet for professionals on pain treatment of the Comprehensive Cancer Centre organisation (VIKC) (both arms).

5. GPs in the Netherlands will be offered a web-based training on the most important aspects of the CPG (intervention arm).

**Figure 2:**
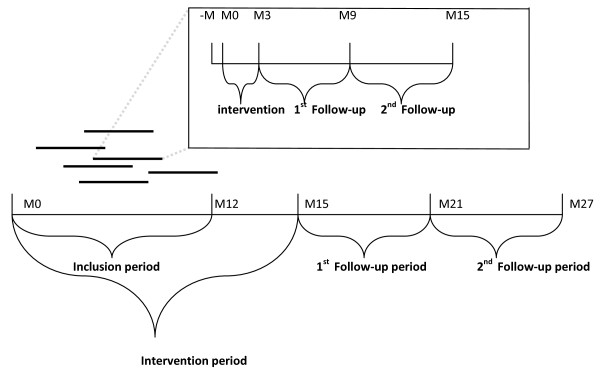
**Overall time chart of the study and per patient (M = month)**. Figure 2 shows the overall time-chart of the study and per patient. Each hospital has a period of twelve months to include 35 patients in the study. The total intervention period of hospitals is fifteen months (per patient twelve weeks). The first follow-up period is six months after the intervention period (M15) and the second, twelve months after the intervention period (M27). Each patient will be included in the study for 15 months.

Follow-up measurements in all hospitals will be conducted to study the long-term effect of the intervention. Regarding the patients recruited in this study, the intention to treat principle will be used (Figure 2).

Furthermore, four times, during a period of one week, transversal measurements will be performed in outpatient clinics of all six hospitals. Pain intensity of all patients who visit the oncology outpatient clinic during that week will be measured (See additional file 1).

### Participant recruitment and inclusion and exclusion criteria

To recruit hospitals, a letter was sent to hospital boards. If the board was willing to cooperate, a meeting with the oncologists and nurse practitioners in oncology was arranged to introduce the study. All hospitals are recruited from the Southeastern region of the Netherlands. Via the hospital boards, professional caregivers, oncologists, and nurses involved in cancer care of the six participating hospitals will be invited to take part. Patients who visit the oncology outpatient clinic will be screened for possible inclusion. Patients will be invited to take part by their medical oncologist or research nurse if they start to experience cancer-related pain.

Overall inclusion criteria for patients are: Diagnosed with cancer; aged 18 years or older; pain intensity of 3 or more on an NRS for the worst pain experienced in the last 24 hours; and having and being familiar with the use of a mobile phone.

Overall exclusion criteria are: Dementia and other severe cognitive disorders; no informed consent; and non-Dutch speaking or writing.

### Intervention

The intervention was based on a pilot study with 13 patients, performed from November 2009 to January 2010, to test feasibility of SMS-IVR. The mean response rate was 62%. A significant reduction of highest pain intensity was found between pre- and post-test (p = 0.018). Pain fluctuated more in patients included in this pilot study than would be expected in patients who will be included in the present study, because only patients in palliative care were included in the pilot study.

Next, we developed a multifaceted intervention with hospital as cluster. Multifaceted interventions are proven to be more effective than single interventions [66,67]. Oncologists and nurses in the hospitals allocated to the intervention condition will be trained in-person, and GPs of patients that take part in the study will be offered a web-based training on the most important aspects of the CPG. Patients in the intervention condition will get SMS-IVR and will receive a personal advice by phone how to reduce their pain if their pain rating is 5 or higher on a NRS of 0 (no pain at all) to 10 (worst pain you can imagine). The research nurse of the hospital, specialised in pain treatment and trained for this project, will provide the personal advice.

The training for oncologists and nurses consists of three one-hour sessions, all given in-person; one main session at baseline, session two at six months, and the final session at 12 months. The first session will include the aim of the study, the main aspects of pain treatment in patients with cancer, pain measurement, and an instruction of the SMS-IVR system in detail. The next two sessions aim to summarise the first session and discuss problems associated with the implementation of the guideline.

**Figure 3:**
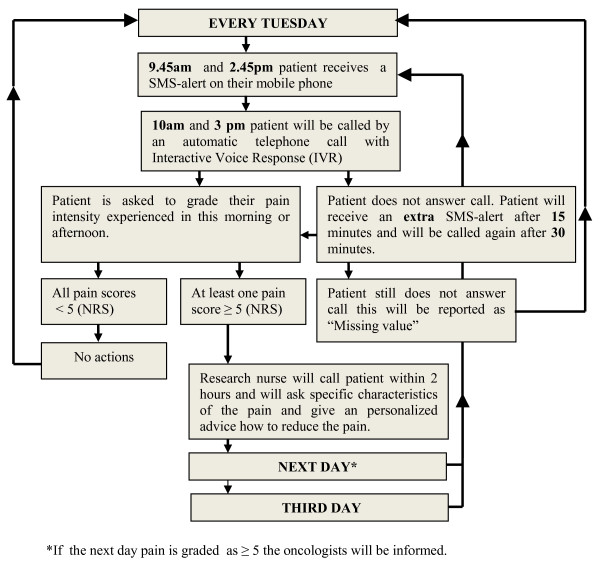
**Workflow SMS alerts**. Figure 3 shows the workflow of the SMS-IVR intervention. Patients receive SMS-IVR minimal once a week (Tuesdays), twice a day, during 12 weeks. SMS alerts are used as a reminder that they will receive an automatic telephone call 15 minutes later with IVR. SMS alerts will be received at 09.45 a.m. and at 2.45 p.m. At 10.00 a.m. and at 3.00 p.m. the patients will be called and invited to rate their pain on a scale of 0 (no pain) to 10(worst pain imaginable). If the highest pain score is 5 or more, the research nurse will contact the patient. If a patient has five or higher on an NRS on Tuesday he/she will again receive two SMS alerts the next day (Wednesday); the procedure will be repeated. For those who still have a pain score of five or higher on Wednesday, the procedure will be repeated again at Thursday. The whole procedure of the SMS-IVR system described in figure 1 will start again the next week at Tuesday.

Figure 3 shows the workflow of the SMS-IVR intervention. Patients receive SMS-IVR minimal once a week (Tuesdays), twice a day, during 12 weeks (Figure 2). SMS alerts are used as a reminder that they will receive an automatic telephone call 15 minutes later with IVR. SMS alerts will be received at 09:45 a.m. and at 2:45 p.m. At 10 a.m. and at 3 p.m. the patients will be called and invited to rate their pain on a scale of 0 (no pain) to 10 (worst pain imaginable). Morning: Rate your pain by choosing a number that best describes your WORST pain in the last 24 hours. Afternoon: Rate your pain at this moment.

If the highest pain score is 5 or more, the research nurse will contact the patient and will ask: 'at what time/period the patients experienced the worst pain and whether the pain limited daily activities?'

Any patient who has 5 or higher on an NRS on Tuesday will again receive two SMS alerts the next day (Wednesday); the procedure will be repeated. For those who still have a pain score of 5 or higher on Wednesday, the procedure will be repeated again at Thursday.

The whole procedure of the SMS-IVR system described in Figure 3 will start again the next week at Tuesday. For the days without SMS, patients will follow the instructions of the research nurse for those days: increase doses with ... when pain is still 5 or higher; keep doses stable at ... when pain is below 5. Thus, at the first day patients will be phoned, because pain score was 5 or higher, the pain treatment protocol will be chosen for the whole week. However, this protocol may be changed by the research nurse when pain intensity remains 5 or higher.

In addition, both patients in the intervention and the control condition will fill in a pain diary on Tuesdays for 12 weeks. They will do this twice each Tuesday, once between 8:00 and 12:00 a.m., and once between 12:00 a.m. and 17:00 p.m.. However, there should be a minimum of five hours between the morning and afternoon measurement. The pain diary reports pain intensity with NRS, use of pain medication, and any side effects of the medication. Patients will also receive a leaflet on cancer pain of the Dutch Cancer Society. In addition, oncologists and nurses will receive a leaflet for professionals on pain treatment of the VIKC.

### Control

Patients in the control condition will also receive a leaflet on cancer pain. These patients will also complete a pain diary on Tuesdays during the 12-week period in the same way as the patients in the intervention condition. In addition, professionals will be offered a leaflet on pain treatment as a summary of the pain management guideline will be offered to GPs in the control condition. This is done to study the effect of the 'active' implementation (interactive web-based training), with the 'inactive' control condition (offering the most important aspects of the guideline as a tool to use in practice).

### Primary and secondary outcome and measurement instruments

The primary outcomes of this implementation study include:

The first primary outcome is the percentage of all patients that visit the medical oncology outpatient clinic with adequate pain therapy/medication. Pain treatment adequacy will be calculated with both the Cleeland's Pain Management Index (PMI)[68] and Ward's variation of the PMI [33]. It is the most used measure for adequate pain treatment [33]. Cleeland's PMI compares the most potent analgesic prescribed, with patient's reported worst pain level on the Brief Pain Inventory (BPI). In addition, Ward's variation of the PMI compares the most potent analgesic used by a patient used with that patient's reported worst level of pain on the BPI. The worst score on the BPI will be determined (1 to 3, mild pain; 5 to 6, moderate pain; and 7 to 10, severe pain), where the absence of pain will be defined as 0, mild pain as 1, moderate pain as 2 and severe pain as 3. The worst score on the BPI is used because it is often used clinically as an indicator for treatment [69]. PMIs will be computed by subtracting the pain level from the analgesic level, ranging from -3 (a patient with severe pain receiving or using no analgesic drug) to +3 (a patient with no pain receiving or using a strong opioid or equivalent). PMI-scores of 0 or higher are considered to be a reflection of adequate pain treatment, whereas negative PMI-scores are considered to reflect inadequate pain treatment.

The second primary outcome is the mean pain intensity of cancer patients, measured with an NRS (SMS alerts and pain diary). The NRS is the most appropriate choice to use in practice for pain intensity [70,71] (see Table 1). The pain dairy is used to obtain additional information (medication use, side effects of medication) and to report pain intensity in the control group.

**Table 1:** Validated patient questionnaires/scales used in this study

Measurement	Validated questionnaires	Time points (M = month)
**Pain intensity**	A. Numeric Rating Scale (NRS) B. Brief Pain Inventory Short form (BP-SF)	A. M0-M3/M9/M15 B. M0/M3/M9/M15

**Multidimensional aspects of pain**	McGill pain questionnaire (MPQ)	M0/M3/M9/M15

**Pain interference with function**	Brief Pain Inventory Short form (BP-SF)	M0/M3/M9/M15

**Adequate pain treatment**	Ward's Pain Management Index (PMI-revised)	M0/M3/M9/M15

**Quality of life**	European Organization for Research and Treatment of cancer Quality of Life Questionnaire- C30 (EORTC QLQC30)	M0/M3/M9/M15

**Neuropathic pain**	Neuropathic Pain Diagnostic Questionnaire (DN4-SF) (first two questions)	M0/M3/M9/M15

**Problems in daily life associated with cancer**	Distress Thermometer (DT)	M0/M3/M9/M15

**Emotions related to cancer**	Hospital Anxiety and Depression Scale (HADS)	M0/M3/M9/M15

**Performance status**	Karnofsky Performance Scale (KPS)	M0/M3

**Self-efficacy for communication about pain with oncologist**	Perceived Efficacy in Patient-Physician Interactions (PEPPI-5)	M0/M3

The secondary outcomes of this study include:

1. Percentage of medical records in which pain of new patients in the outpatient oncology clinic is registered with a validated instrument, such as the NRS or visual analogical scale (VAS). These data will be collected retrospectively via medical records.

2. Quality of life of patients with The European Organization for Research and Treatment of Cancer Core Quality of Life Questionnaire Quality of life (EORTC QLQ-C30) questionnaire [72]. EORTCQLQ-C30 will be used to measure quality of life [72].

3. Knowledge of medical oncologists and nurses of the content of the guideline with a self-developed and pilot-tested knowledge questionnaire and vignette study. This knowledge questionnaire and vignette study are based on the recommendations in the guideline, with input from specialists in pain treatment and GPs.

4. Pain intensity and impact of pain on daily activities will be measured with the Brief Pain Inventory Short Form (BPI-SF) [73]. This questionnaire consists of four questions whereby pain intensity is rated on an 11-point numerical scale (NRS) raging from 0 (no pain) to 10 (worst pain ever).

5. Insight in the multidimensional aspects of pain with the Short-form McGill Pain Questionnaire [74]. To measure sensory, affective, and evaluative qualities of pain the McGill Pain Questionnaire Dutch version (MPQ-DV) will be used [75].

6. Performance status of patients will be measured with the Karnofsky scale [76]. It is based on the assessment by the oncologist of the patient's ability to perform usual daily activities.

7. We will identify neuropathic pain by using the two first questions of the Douleur Neuropathic 4 questions questionnaire, short form (DN4-SF) [77].

8. To assess multidimensional problems (work, family, *et al*.) related to cancer the distress thermometer (DT) will be used [78].

9. Prevalence of anxiety and depression will be measured with the Hospital Anxiety Depression Scale HADS [79].

10. Patients' experiences with the SMS-IVR system will be assessed with semi-structured interviews.

11. Self-efficacy for communicating about pain with oncologists will be assessed with the mean response to the five items in the Perceived Efficacy in Patient-Physician Interactions scale (PEPPI-5), with the wording of the items modified to refer to communication about pain with oncologists [80] (Table 1).

### Sample size

Sample size calculations of the present study with three clusters of two hospitals, are based on the expected effect of the intervention on the PMI. However, the present study is the first investigating the effects of using an SMS-IVR system in cancer pain management. Several studies show that adequate pain relief can be achieved in 70 to 90% of patients with cancer [44-47]. To achieve this, the present study aims to find out whether our implementation strategy reduces the negative PMI from 42% [6] to 20% [44-47] of all cancer patients visiting the outpatient clinic.

To detect a difference with 80% power (alpha = 0.015), we need 90 patients per condition. Accounting for clustering resulted in an intraclass correlation coefficient (ICC) of 0.015. Based on the ICC and three hospitals per condition, we need 30 patients per hospital. Taking into account a dropout rate of 15%, we need 35 patients per hospital, for a total of 210 patients.

### Cluster randomisation of clinics

Clusters of hospitals will be determined based on number of beds and number of medical oncologists to increase comparability of hospitals and to reduce contamination [64]. Of each pair, one hospital will be randomly allocated to the intervention condition and the other to the control condition. Randomisation took place after all hospital boards and medical oncologists had agreed to participate. Next, an independent statistician allocated to the intervention or control condition based on clusters by selecting three closed envelopes (Figure 1). Patients will be invited to take part by their medical oncologist or research nurse.

### Statistical analysis

To measure the effect of the implementation the PMI and NRS will be used and tested with general linear model analysis of variances (GLM ANOVA) repeated measures. Qualitative content analysis will be used to analyse the results of the focus group discussions and to analyse the interviews to evaluate the SMS alert intervention. Qualitative analysis will be supported by the use of the Atlas.ti software programme.

Data collected via SMS-IVR will be analysed for descriptive data: how did pain scores change and fluctuate in the whole period, what actions were taken by the research nurse, and did this intervention help the patients to manage pain? Subgroup analysis will be conducted. Differences in subgroups of the effectiveness of the intervention are expected. Subgroups will be classified by: age, gender, race, education, performance status, and classification of malignant tumors (TNM stage). The most recent version of SPSS will be used to perform the statistical analysis.

### Qualitative data collection

Many studies explored barriers in pain management of patients with cancer and professional caregivers in different countries. However, this has never been done in the Netherlands. Therefore, four focus group interviews will take place to explore barriers and incentives about cancer pain management with respectively: patients with cancer, oncologists, nurses and GPs. Focus groups offer an opportunity to obtain significant insight regarding the experiences, observations, and opinions of members of that group [81].

In addition, semi-structured interviews by phone focused on patient empowerment will be used to evaluate the SMS-IVR intervention. Ten randomly selected participating patients per hospital will be interviewed. The aim of these interviews is to shed light on the results of the intervention and the effect on patient empowerment.

### Retrospective analysis

To investigate how and how frequently pain has been reported in medical records retrospective analysis will be performed for the year 2010 (two years after the guideline has been published). Thirty-six medical records per hospital (the first three of each month) of oncology patients who came for their first consultation at the outpatient clinic will be obtained. Retrospective analysis of medical records will be repeated after the intervention period.

### Additional data

Data on patients characteristics will be obtained from medical records: patient identification code, date of diagnosis, gender, age, postal code, marital status, primary cancer type, secondary cancer, history of cancer treatment, present treatment, cancer exact location, TNM stage cancer, and pain medication. Retrospective data of surgery and other cancer treatment during intervention period, and hospital admission(s) (number, length and indication) will also be analyzed. Other data will be obtained via a patient questionnaire including questions about: SMS use, education level, and experiences with present pain treatment.

### Ethical considerations

The study has been approved by the Medical Ethics Committee (CMO) of the Radboud University Nijmegen Medical Centre (METC protocol number 2011/020) (See additional file 2). The Dutch Cancer Society (KWF) approved the research protocol, which has been registered by the Dutch Trial Register (NTR2739). This study has also been registered by the local ethical committees of each hospital. Anonymity of every patient is guaranteed. Patients have to sign an informed consent before start of the intervention.

## Discussion

This implementation study will be the first RCT to study the use of SMS- IVR to collect data on cancer pain. Furthermore, this study is innovative in the active involvement of oncologists, nurses, GPs, and patients with cancer from guideline development to the implementation of the guideline. SMS and/or IVR have never been used before to assess pain in patients with cancer. Using SMS-IVR as a reminder and as a tool to collect data on pain scores is an innovative and promising method [55]. It does not interfere with the patient's daily activities, because SMS has become part of daily life [59]. Pain can be measured systematically at any location with SMS-IVR, the patient can prepare himself (reminder before the actual call), can grade his pain two times a day without much effort and time investment, and, if necessary, can be treated earlier than in usual care.

The use of SMS alerts and mobile phone in the present study may be a way to encourage patient empowerment, because the patient's role in their pain management becomes more active. Another way to describe this is that it may increase patient participation. Whether the use of SMS alerts and mobile phones with IVR to report pain in patients with cancer may increase patient empowerment or patient participation can be questioned. Patient empowerment is a commonly used term within healthcare, but there is little consensus regarding its definition [82]. In this intervention, the patient is not able to report pain at any time. However, the SMS alert may increase the feeling of having control. Therefore, the SMS alert intervention increases patient-participation and may increase the feeling of having control over one's life. In this way the SMS alert intervention may encourage patient empowerment.

In addition, our study will show possible barriers in SMS-IVR use for pain reporting in patients with cancer. This has never been done before. One of the possible barriers accounted for in the present study is asking too often about cancer pain and this could be experienced as confrontation with their disease. However, nothing is known yet about a proper frequency to ask patients about their pain. In the present study, patients will receive a weekly SMS alert twice a day. In the pilot study, patients received SMS alerts four times a week for four weeks. To achieve a similar response rate and compliance as was achieved in the pilot study, the frequency of SMS alerts has been reduced in the present study. It has been reduced to once a week if there is no pain and to maximal three times a week if pain remains present because the intervention period is three times as long. Asking patients about pain improves insight in pain intensity of professionals and it increases registration of pain [83,84]. Asking about pain in itself can reduce pain intensity [84]. Therefore, using SMS-IVR as a way to systematically measure pain is expected to reduce pain intensity.

Apart from the SMS-IVR, a pain dairy is necessary to obtain data on pain intensity in the control group and additional information in both control and intervention group. Asking about pain by measuring pain intensity with a pain diary in itself can reduce pain intensity [84]. Therefore, we expect that pain intensity difference between the intervention and the control group will be smaller. However, the possibility of earlier treatment is restricted to the intervention group. We expect an increase in motivation of patients to take part in the control condition and higher compliance during the study than without the pain dairy. However, because patients are expected to be more motivated to participate when SMS alerts are offered to them, this may cause selection bias. However, it was not possible to randomise at patient level, because of the multifaceted intervention. Oncologists and nurses should be trained before inclusion of patients.

This study protocol shows that the present study is the first to use SMS alerts as a reminder in patients with cancer and mobile phones with IVR to collect data on cancer pain. Furthermore, this study is innovative in the active involvement of oncologists, nurses, GPs, and patients with cancer from guideline development to the implementation of the guideline. If the implementation proves to be effective, it can be considered for use in other hospitals to increase percentage of patients with cancer that receive adequate pain therapy and to reduce pain intensity in patients with cancer. If SMS-IVR proves to be an acceptable and useful method for patients and medical professionals with cancer to report their pain, it can be considered for use of data collection to report pain. Therefore, the SMS alert intervention increase patient participation and may increase the feeling of having control over one's life. In this way the SMS alert intervention may encourage patient-empowerment.

## Competing interests

The authors declare that they have no competing interests.

## Authors' contributions

All authors contributed to the design of the study. NtB and CB are the principal researchers. NtB was responsible for writing this paper. All other authors scrutinized the manuscript. EY coordinates the study. KV and MVD supervise this study. All authors have read and approved the final manuscript.

## Authors information

NtB is the principal investigator in this study. EY combines experience in quality of care research (indicator development and implementation, improving quality of care, changing behaviour of professionals) with experience in research in pain and palliative care. CB, an anesthesiologist and pain therapist, was involved in the development of the guideline and is also a principal investigator in this study. KV is head of the Knowledge Center Of Pain And Palliative Medicine, and a medical specialist in pain control and palliative medicine. He was chairman of the developmental process of the guideline 'pain in cancer patients', and has a national and international respected knowledge and experience in the field of pain diagnosis and therapy. MVD has an extensive record of experience in research in palliative care, quality of care research, and in implementation research.

## Supplementary Material

Additional file 1 **Consort checklist**. Checklist of items to include when reporting a cluster randomised trial. Click here for file

Additional file 2 **Medical Ethics Committee (CMO)**. Approval letter of Medical Ethics Committee. Click here for file

Additional file 3 **Finance**. Funding letter by KWF, Dutch Cancer Society and Bergh in het Zadel (Dutch Association that funds research). Click here for file
