# Supplementary material for: Rationale, design, and implementation protocol of the Dutch clinical practice guideline Pain in patients with cancer: a cluster randomised controlled trial with short message service (SMS) and interactive voice response (IVR)
Source: Implement Sci. 2011 Dec 6;6:126. doi: 10.1186/1748-5908-6-126 (PMC3248867; doi:10.1186/1748-5908-6-126)
Supplement: Additional file 1 — Consort checklist. Checklist of items to include when reporting a cluster randomised trial. [file 1748-5908-6-126-S1.PDF]

Checklist of items to include when reporting a cluster randomised trial

* = addition to CONSORT *Modifications to checklist in italics*			
PAPER SECTION and topic	Item	Descriptor	Reported on Page No.
TITLE & ABSTRACT	1*	How participants were allocated to interventions (e.g., "random allocation", "randomised", or "randomly assigned"), *specifying that allocation was based on clusters*	Page 1 & 2
INTRODUCTION Background	2*	Scientific background and explanation of rationale, *including the rationale for using a cluster design.*	Page 3-5
METHODS Participants	3*	Eligibility criteria for participants *and clusters* and the settings and locations where the data were collected.	Page 7
Interventions	4*	Precise details of the interventions intended for each group, *whether they pertain to the individual level, the cluster level or both,* and how and when they were actually administered.	Page 8-9
Objectives	5*	Specific objectives and hypotheses, *and whether they pertain to the individual level, the cluster level or both.*	Page 6
Outcomes	6*	Report clearly defined primary and secondary outcome measures, *whether they pertain to the individual level, the cluster level or both,* and, when applicable, any methods used to enhance the quality of measurements (e.g., multiple observations, training of assessors).	Page 9-11
Sample size	7*	How *total* sample size was determined *(including method of calculation, number of clusters, cluster size, a coefficient of intracluster correlation (ICC or k), and an indication of its uncertainty*) and, when applicable, explanation of any interim analyses and stopping rules.	Page11
Randomisation.			Page 11
Sequence generation	8*	Method used to generate the random allocation sequence, including details of any restriction (e.g., blocking, stratification, *matching*).	
Allocation concealment	9*	Method used to implement the random allocation sequence, *specifying that allocation was based on clusters rather than individuals and* clarifying whether the sequence was concealed until interventions were assigned.	Page 11
Implementation	10	Who generated the allocation sequence, who enrolled participants, and who assigned participants to their groups.	Page 11
Blinding (Masking)	11	Whether or not participants, those administering the interventions, and those assessing the outcomes were blinded to group assignment. If done, how the success of blinding was evaluated.	Page 6 first sentence study design.
Statistical methods	12*	Statistical methods used to compare groups for primary outcome(s) *indicating how clustering was taken into account*; methods for additional analyses, such as subgroup analyses and adjusted analyses.	Page 12
RESULTS Participant flow	13*	Flow of *clusters and* individual participants through each stage (a diagram is strongly recommended). Specifically, for each group report the numbers of *clusters and* participants randomly assigned, receiving intended treatment, completing the study protocol, and analyzed for the primary outcome. Describe protocol deviations from study as planned, together with reasons.	n/a study protocol
Recruitment	14	Dates defining the periods of recruitment and follow-up.	n/a study protocol
Baseline data	15*	Baseline information for each group *for the individual and cluster levels as applicable*	n/a study protocol
Numbers analyzed	16*	Number of *clusters and* participants (denominator) in each group included in each analysis and whether the analysis was by "intention-to-treat". State the results in absolute numbers when feasible (e.g., 10/20, not 50%).	n/a study protocol
Outcomes and Estimation	17*	For each primary and secondary outcome, a summary of results for each group measures *for the individual or cluster level as applicable,* and the estimated effect size and its precision (e.g., 95% confidence interval) *and a coefficient of intracluster correlation (ICC or k) for each primary outcome.*	n/a study protocol

Ancillary analyses	18	Address multiplicity by reporting any other analyses performed, including subgroup analyses and adjusted analyses, indicating those pre-specified and those exploratory.	n/a study protocol
Adverse events	19	All important adverse events or side effects in each intervention group.	n/a study protocol
DISCUSSION<br>Interpretation	20	Interpretation of the results, taking into account study hypotheses, sources of potential bias or imprecision and the dangers associated with multiplicity of analyses and outcomes.	Page 14
Generalisability	21*	Generalisability (external validity) *to individuals and/or clusters (as relevant)* of the trial findings.	Page 15
Overall evidence	22	General interpretation of the results in the context of current evidence.	n/a study protocol
