# Supplementary material for: Rationale, design, and implementation protocol of the Dutch clinical practice guideline Pain in patients with cancer: a cluster randomised controlled trial with short message service (SMS) and interactive voice response (IVR)
Source: Implement Sci. 2011 Dec 6;6:126. doi: 10.1186/1748-5908-6-126 (PMC3248867; doi:10.1186/1748-5908-6-126)
Supplement: Additional file 2 — Medical Ethics Committee (CMO). Approval letter of Medical Ethics Committee. [file 1748-5908-6-126-S2.PDF]

UMC St Radboud

Universitair Medisch Centrum

Instituut Waarborging Kwaliteit en Veiligheid
Commissie Mensgebonden Onderzoek
regio Arnhem - Nijmegen

Huispost 578
Postbus 9101
6500 HB Nijmegen

UMC St Radboud Centraal, route 578
Geert Grooteplein 10

T (024) 361 31 54
F (024) 361 01 48

cmo@iwkv.umcn.nl
www.umcn.nl
www.cmoregio-a-n.nl

578

Mw. N.D. te Boveldt, MSc,
Anesthesiologie, Huispost 630

Datum 20 januari 2011
Ons kenmerk CD/CMO 0110

Titel: Implementatie van de richtlijn pijn bij kanker
Registratienummer: 2011/020

Geachte mevrouw Te Boveldt,

In antwoord op uw e-mail d.d. 17 januari 2011 bericht ik u namens de CMO als volgt.

Met inachtneming van hetgeen dienaangaande in de Wet medisch-wetenschappelijk onderzoek (WMO) is bepaald, oordeelt de commissie dat het onderzoek niet valt aan te merken als een onderzoek dat valt onder de reikwijdte van WMO. De CMO als erkende commissie is derhalve niet bevoegd het onderzoek te beoordelen.

Dit betekent dat het onderzoek kan worden uitgevoerd zonder beoordeling door de CMO.

Dit oordeel is gebaseerd op de volgende documenten:

- de e-mail van N.D. te Boveldt, MSc, d.d. 17 januari 2011, waarbij gevoegd:
- verzoekschrift beoordeling onderzoeksprotocol "Pijnsein" d.d. 14 januari 2011
- samenvatting van het onderzoeksvoorstel
- vragenlijst *knelpunten pijnbehandeling bij kanker voor patiënten*

Wellicht ten overvloede attendeert de commissie u erop dat de omstandigheid dat het onderzoek zonder positief oordeel van de CMO kan worden uitgevoerd, uiteraard niet betekent dat u zich bij het uitvoeren van het onderzoek niet heeft te houden aan de regels die gelden voor het uitvoeren van niet-WMO-plichtig onderzoek.

Ik vertrouw erop u met deze brief van dienst te zijn en namens de commissie wens ik u succes met de uitvoering van het onderzoek.

Met vriendelijke groet,

Dr. M.J.J. Prick, vice-voorzitter b.a. M. [illegible] secretaris
