# Supplementary material for: Rationale, design, and implementation protocol of the Dutch clinical practice guideline Pain in patients with cancer: a cluster randomised controlled trial with short message service (SMS) and interactive voice response (IVR)
Source: Implement Sci. 2011 Dec 6;6:126. doi: 10.1186/1748-5908-6-126 (PMC3248867; doi:10.1186/1748-5908-6-126)
Supplement: Additional file 3 — Finance. Funding letter by KWF, Dutch Cancer Society and Bergh in het Zadel (Dutch Association that funds research). [file 1748-5908-6-126-S3.PDF]

KWF KANKER BESTRIJDING

Postbus 75508
1070 AM Amsterdam

T (020) 570 05 00
F (020) 675 03 02
Giro 26000
www.kwfkankerbestrijding.nl

CONTACT
Afdeling Subsidies
E subsidies@kwfkankerbestrijding.nl
T (020) 57 00 432

Radboud Universiteit Nijmegen
T.a.v. Prof.dr. K.C.P. Kris Vissers
Medical Sciences
Geert Grooteplein-Zuid 8
6525 GA NIJMEGEN

BEDRIJFSEENHEID PPP UMCN			
NUMMER 10.318		DATUM ONTVANGST 9 dec. 2010	
BEHANDELEN DOOR:		KOPIE AAN:	
	K.V.	Y.E.	Simon Broeck.
		Kees Besse	

Datum 6 december 2010
Onderwerp Project adoptie/4724
Pagina 1/1
Ons kenmerk PA-Wetenschapper informeren-KUN 2010-4724
Uw kenmerk -

Geachte prof. Vissers ,

Met de financiële steun van KWF Kankerbestrijding zal door u het project : KUN 2010-4724, getiteld 'Implementing the Dutch guideline "Pain in Cancer Patients"' worden uitgevoerd. Conform hetgeen is vermeld onder punt 29 van de subsidievoorwaarden hebben wij uw project ingezet bij onze fondsenwervende activiteiten.

Wij zijn verheugd u te kunnen melden dat Stichting Berg in het Zadel financieel wil bijdragen aan uw project. KWF Kankerbestrijding is hiermee bijzonder ingenomen. Bij het toezeggen van subsidie aan u is KWF Kankerbestrijding er vanuit gegaan dat de daartoe benodigde financiële middelen in de loop van het begrotingsjaar geworven zouden worden. De bijdrage van Stichting Bergh in het Zadel is geen additionele bijdrage van KWF Kankerbestrijding, maar een onderdeel van de subsidie. Voor vragen hierover verwijzen we u naar dr. Gijs Boerrigter.

Wij verzoeken u vriendelijk eventueel een bijdrage te leveren in de presentatie van uw wetenschappelijk onderzoek. Dit kan onder andere betekenen dat u bijvoorbeeld namens KWF Kankerbestrijding een symbolische cheque in ontvangst zal nemen. Te zijner tijd nemen wij tijdig contact hierover met u op.

Wij kijken uit naar een succesvolle samenwerking!

Met vriendelijke groet,

Malika el Hamami
coördinator subsidies

Cc. Dr. Gijs Boerrigter, Hoofd Programma Onderzoek
